# A holistic study of neonicotinoids neuroactive insecticides—properties, applications, occurrence, and analysis

**DOI:** 10.1007/s11356-019-06114-w

**Published:** 2019-09-13

**Authors:** Bogusław Buszewski, Małgorzata Bukowska, Magdalena Ligor, Irena Staneczko-Baranowska

**Affiliations:** 1 Chair of Environmental Chemistry and Bioanalytics, Faculty of Chemistry, Nicolaus Copernicus University, 7 Gagarina Str., 87-100 Torun, Poland; 2 Interdisciplinary Centre of Modern Technologies, Nicolaus Copernicus University, 4 Wileńska Str., 87-100 Torun, Poland; 3 Department of Inorganic, Analytical Chemistry and Electrochemistry, Faculty of Chemistry, Silesian University of Technology, 7 M. Strzody Str., 44-100 Gliwice, Poland

**Keywords:** Neonicotinoids, Applications, Properties, Analytics, Bee life

## Abstract

Among pesticides and foliar sprays involved in the treatment of seed, soil, and grass, also to crops, an important group is neonicotinoids. Neonicotinoid pesticides present similar properties with nicotine, but the mentioned compounds are less harmful for humans. Nevertheless, neonicotinoids are poisonous to insects and some invertebrates, which can act against insects’ central nervous system, leading to their death. Moreover, neonicotinoids can affect the reproduction, foraging, and flying ability of honeybee and other insects including pollinators. In the present study, some neonicotinoids, such as imidacloprid, acetamiprid, clothianidin, thiacloprid, and thiamethoxam together with their toxic effects, have been presented. The Environmental Protection Agency (EPA) classifies these neonicotinoids as II and III class toxicity agents. Due to accumulation of these pesticides into the pollen of treated plants, especially due to their toxic effects against pollinators, the consequences of the occurrence of these insecticides have been discussed. Analytical aspects and methods involved in the isolation and determination of this class of pesticides have been presented in this contribution.

## Introduction

The increasing use of chemical products in different spheres of life not only brings benefits for the humanity but also presents a large number of threats against the environment and in consequence to human health. However, without transformations that occurred thanks to chemical industry, the progress of civilization would have been much slower. Huge progresses were made in agriculture, particularly regarding different practices using technology and agrichemicals.

Nevertheless, the products’ quality depends not only on soil and climate but also on the methods of fertilizing and cultivation. Thus, growing crop plants requires precautions that can guarantee abundant high-quality yield. Most yield gains are due to genetic improvements and fertilizer addition, not to pesticides. Moreover, herbicides certainly help to reduce competition between cultivated plants and unwanted weeds and help in yield increasing (Lechenet et al. 2017). Consequently, very often the quality is not solely dependent on nature, and can be strongly influenced by chemical substances used for plant growing and protection. Their use is more than once necessary as insects, diseases, fungi, and weeds may attack and compete the cultivated plants by stealing nutrients, moisture, and sunlight. Therefore, some farmers have been using plant protection agents, including pesticides, but this is with opposition of organic farmers, who promote the total elimination of pesticides. Nevertheless, pesticides are natural or synthetic substances commonly used into control of harmful or undesirable organisms both in agriculture and in homes. Their use in forest protection and of bodies of water is known. Pesticides are represented by a large group of chemical compounds with a broad range of actions. These classifications are available based on firstly at the target organism to control (insecticides, herbicides, fungicides or biocides), and second the physiological acting mechanism within each of those groups, which is usually related to particular chemical structures. A major inconvenience in the use of pesticides is the influence of their residues on the environment. Generally, they are xenobiotics, which can remain in various elements of the environment, presenting toxicological properties for living organisms. Paracelsus, the founder of toxicology, pointed out as early as the 15th century that in any poison the most important is the amount: the Latin phrase *sola dosis facit venenum* means “the dose makes the poison.” Therefore, we ought to support both the tendency to limit quantity of toxic substances used and elimination of products which pose a significant danger to ecosystems (Dobrzański et al. 2017). Nevertheless, another important factor that determines the dose that results in chronic toxicity is the time of exposure or contact time of living organism with a toxin. Let us not forget that the duration and long-term release of some toxic substances is also dangerous. Therefore, completing, we can state that dose and time of exposure make the poison (Tennekes 2017).

**Fig. 1:**
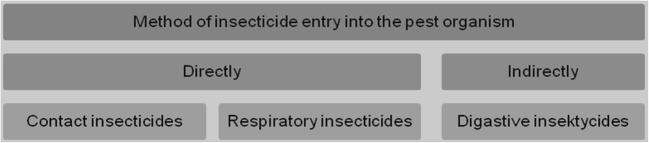
Routes of entry of active substances of insecticide products into the organism of a pest

Dependent by the mechanism of action in a plant, we can distinguish between contact (surface) insecticides—which remain and act on the external plant surface—and systemic insecticides that enter into the plant system and are transported with sap to the whole plant parts. Even if the mentioned components are beneficial for plants, they are poison for insects which are living around and feeding from plants (Bonmatin et al. 2015). Insecticides may be divided into two groups (Fig. 1) depending on the penetration of the active substance into the pest, either directly (via the exoskeleton or the respiratory system) or indirectly (with a poisoned plant). The first group includes contact and respiratory insecticides while the second one, digested ones.

Contact insecticides are soluble in lipids, which mean that can easily penetrate the insect’s body, causing loss of coordination, convulsions, and contractions of the whole body, leading finally to death. Generally, the insecticides can act against insects’ respiratory system in all their growing stages except eggs’ phase. The penetration way of the insect’s body is as a vapor or gas which is inhaled which blocks the activity of respiratory enzymes. Other insecticides act when they are ingested and poisoned plant matter gets into the insect alimentary tract. These types of insecticides can be used as a soil formulation, from which through the roots they get to the aerial parts of the plant (Das 2013).

**Fig. 2:**
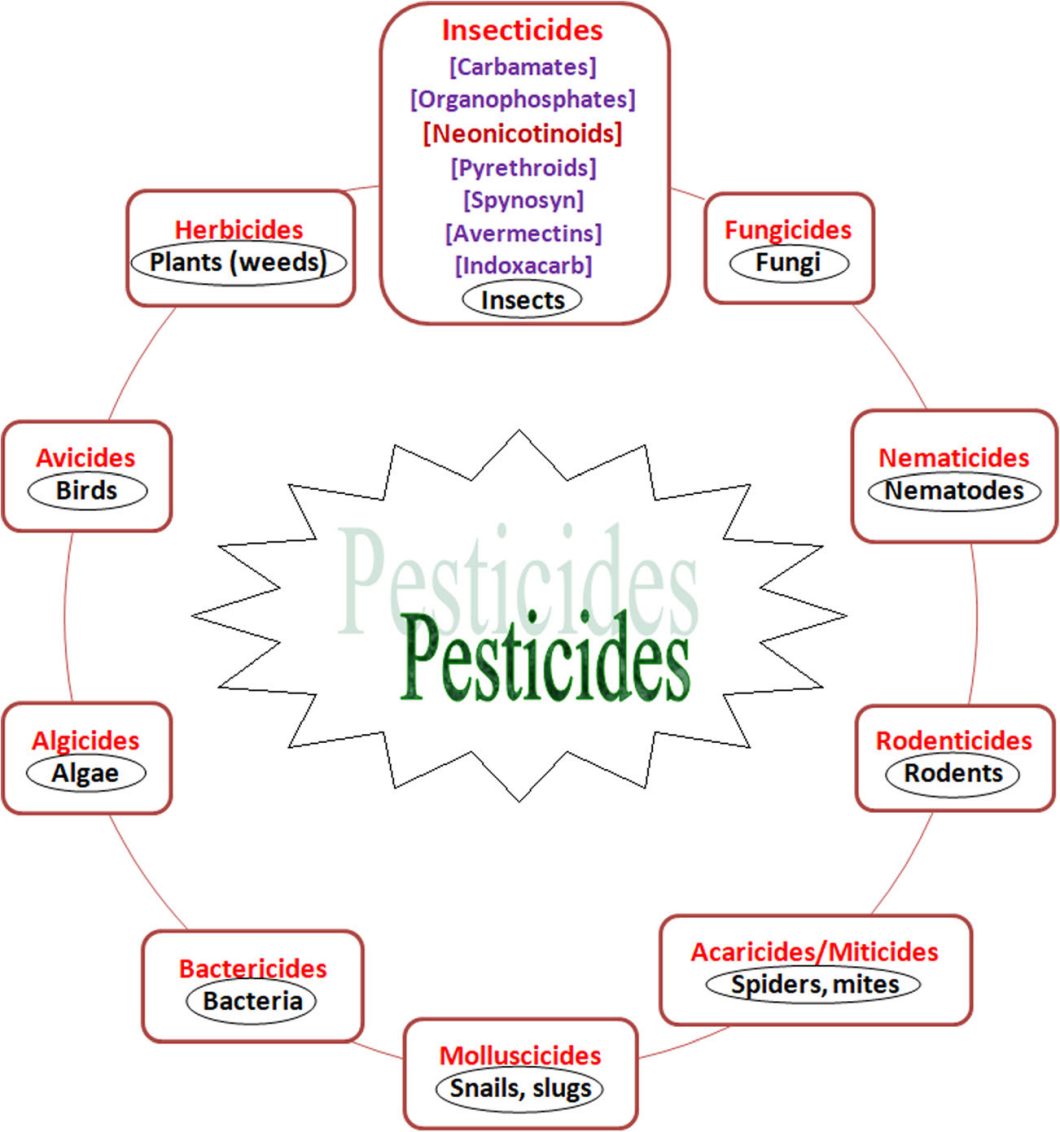
Classification of pesticides according to use

However, the pesticides and plant protection products are frequently classified according to their main use. As chemical products are used to protect plants in gardens and fields, they have a large spectrum of action modes. It is important to know what threats their use may pose (e.g., disruption of plant growth) and the specific ways individual formulations operate (Bateman et al. 2016; Zilberman and Millock 1997). In general, the products currently in use ought to meet some of the criteria before they are authorized for marketing. Risks to human health and the impact on the environment are important, which increases the likelihood of cancellation of selected pesticides (Cropper et al. 1992). It is extremely important that pesticides and plant protection products are to be both effective and safe for organisms inhabiting a given environment. Another area to focus on is the life span of the used formulations because when they are persistent and their residue remains, they significantly endanger the environment. With regard to modes of action, pesticides can be classified into several groups and subgroups presented in Fig. 2 (Bateman et al. 2016). An important type of pesticides is represented by neonicotinoids, the main subject of this study.

Neonicotinoids belong to the group of active compounds against a broad spectrum of crop pests; therefore, they are important for economic reasons. We also consider the impact on the environment, insects, pollinators, small aquatic mammals, birds, amphibians, reptiles, and fish. An important part of investigations is the development of sample preparation methods as well as qualitative and quantitative analysis; that reason analysis of neonicotinoids has been a crucial part of the presented work.

## Neonicotinoids—a new insecticide class

In the beginning of 1990s, a new group of active compounds was introduced, including neonicotinoid compounds (e.g., imidacloprid, acetamiprid, nitrosoguanidine, dinotefuran, clothianidin, thiacloprid, and thiamethoxam); all of them are insecticides (Frederickson et al. 2016; Simon-Delso et al. 2015). In contrast to the previously used agents, neonicotinoids present the advantage that they can be used in smaller doses. In time, a substantial progress was developed in the production of pesticides as well as in other plant defender products. In the beginning, the recommended doses of preparations with substances belonging to various chemical groups were lowered. For example, the recommendation for DDT and other chlorines was to use up to 1.5 kg of substance per hectare, organophosphate insecticides were effective at half that dose, and the products containing neonicotinoids are used at the doses below 20 g of substance per hectare (Michalcewicz 1995; Reynoso et al. 2019; Wood and Goulson 2017).

Chemically, neonicotinoids are related to nicotine. Nicotine itself has insecticidal properties and in the past it was used for many years as infusions meant to eliminate some species of pests. Good results in controlling aphids and various greenhouse crop pests were achieved by fumigating greenhouse by burning nicotine, mainly used as nicotine sulfate. However, nicotine is toxic also to mammals, a reason why it ceased to be utilized in plants’ protection. In reality, the nicotine lethal dose is higher for flies than for rats. Research studies have been developed to transform nicotine into substances that are safer but still highly efficient in controlling pests. Nicotine has been applied in agriculture, not only in the form of sulfate but also in extracts that were very effective to control most kinds of insects. The recent ban of nicotine (nicotine sulfate) for agricultural purposes is not thanks to its toxicity to humans, but rather to resistance process developed by insects to this substance (Hayes 1982). Moreover, the commercialization of neonicotinoids is more profitable for the companies that make them than nicotine—this natural compound is easily extracted from tobacco leaves at lower cost. In this way, neonicotinoids gained increasing popularity; they can be easily dissolved in water and slowly break down in the soil, so they are easily absorbed by plants and provide protection during plant growth (Frederickson et al. 2016; Yamamoto et al. 1995).

Development of neonicotinoids was started in the 1990s by Bayer (Simon-Delso et al. 2015). The Bayer company developed for the first time a commercial neonicotinoid, imidacloprid, which in the end of 1990s was already used on a large scale. In beginning of 2000s, two new neonicotinoids were introduced to the general market—clothianidin and thiamethoxam. Currently, the majority of crops (mainly corn and soy) are treated with one neonicotinoid and fungicidal products (Jeschke et al. 2011; Yang et al. 2014).

## Structure and properties of neonicotinoids

The properties and certain physicochemical parameters of selected neonicotinoids can be observed in Table 1.

**Table 1:** Physical properties of neonicotinoids

	Thiamethoxam	Clothianidin	Imidacloprid	Acetamiprid	Thiacloprid
Systematic nomenclature	3-[(2-Chloro-1,3-thiazol-5-yl)methyl]-5-methyl-*N*-nitro-1,3,5-oxadiazinan-4-imine	1-(2-Chloro-1,3-thiazol-5-ylmethyl)-3-methyl-2-nitroguanidine	*N*-{1-[(6-chloro-3-pyridyl)methyl]-4,5-dihydroimidazol-2-yl}nitramide	*N*-[(6-chloro-3-pyridyl)methyl]-*N*′-cyano-*N*-methyl-acetamidine	{(2Z)-3-[(6-Chloropyridin-3-yl)methyl]-1,3-thiazolidin-2-ylidene}cyanamide
Summary formula	C_8_H_10_ClN_5_O_3_S	C_6_H_8_ClN_5_O_2_S	C_9_H_10_ClN_5_O_2_	C_10_H_11_ClN_4_	C_10_H_9_ClN_4_S
Absorbance	244 nm 253 nm 254 nm	266 nm 270 nm	266 nm 270 nm	244 nm 245 nm	242 nm 244 nm 245 nm
Molecular weight	291.71 g/mol	249.68 g/mol	255.66 g/mol	222.68 g/mol	252.72 g/mol
CAS	153719-23-4	210880-92-5	138261-41-3	135410-20-7	111988-49-9
Characteristic	White-beige, odorless, crystalline powder	White powder	Solid	Powder	Solid, whitish, no odor

Like any pesticide group, several chemical subgroups can be distinguished within the neonicotinoids based on their molecular structures. According to the action mechanism, neonicotinoids are the same because they all act as agonists of the on the nicotine receptors of acetylcholine (nAChRs) because they have the same active moiety (Moffat et al. 2016; Pisa et al. 2017; Simon-Delso et al. 2015; Wood and Goulson 2017). Neonicotinoids are not a uniform chemical group, which is reflected by their susceptibility to decomposing in the soil, their metabolism in an insect’s organism, and their impact on bees. Nevertheless, the cyano-substituted compounds have lower toxicity to bees than the other neonicotinoids. This is given by a detoxification mechanisms that occur for which we have no explanation so far. However, cyano-substituted neonics are as toxic to aquatic insects as the nitroguanidine neonics (Morrissey et al. 2015).

**Fig. 3:**
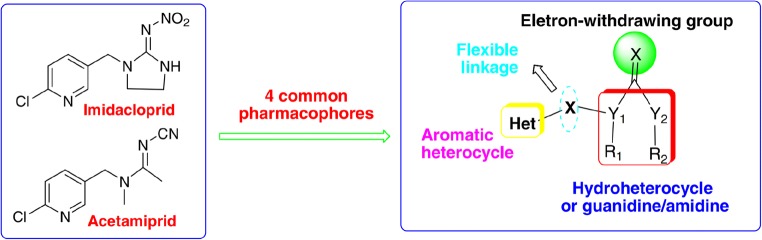
Models of positions of atoms and functional groups. Original substances: imidacloprid and acetamiprid, according to ref. Yang et al. (2014)

As for chemical structure, different classifications of neonicotinoids have been applied. The basis for classification of these aromatic heterocyclic compounds can be the presence of conjugated bonds. Neonicotinoids are hydroheterocyclic guanidines/amidines, and they posses active substituents (Fig. 3) (Yang et al. 2014).

Chemical structures of neonicotinoids created in the last decades have four common elements: (1) aromatic heterocyclic group, (2) elastic bonds, (3) hydroheterocyclic, or guanidine/amidine groups, and (4) electron withdrawing group. Besides, new neonicotinoid derivatives are continuously created by modifying the structures of the mentioned compounds by introducing a sulfonamide functional group or its cyclical equivalent instead of cyano- or nitroguanidine/amidine group (sulfonamide neonicotinoids, e.g., sulfoxaflor). The sulfonamide derivatives may show significant activity in insect and acari control, but a change of substitutes can result in great disparities in strength. Individual insecticides may vary in strength and way of action due to a combination of factors like the number of substitutions, elasticity, or participation of free electrons (Yang et al. 2014).

**Fig. 4:**
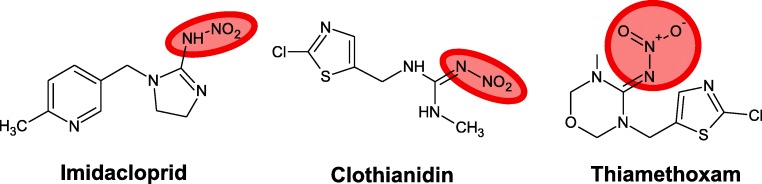
Neonicotinoids of the nitroguanidine group, toxic to bees

A simpler division of these subclasses is also possible, with just two types: nitroguanidine and cyanoamidine. Neonicotinoids of the nitroguanidine type contain inside their structure N-nitro groups, which contain oxygen atoms; consequently, these particles are much more polar and reactive. This group includes imidacloprid, thiamethoxam, and clothianidin (Fig. 4). Due to its structure, clothianidin can be counted among the substances most toxic to honey bees (Pisa et al. 2015).

**Fig. 5:**
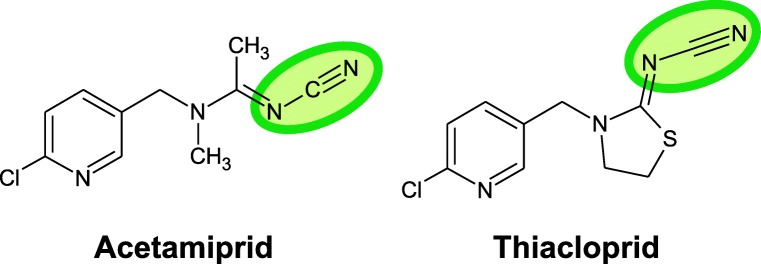
Neonicotinoids of the cyanoamidine group, non-toxic to bees

Neonicotinoids of the cyanoamidine type instead of nitro groups contain in their particles cyanoamidine groups, which do not include oxygen atoms, and thus they are less polar and less reactive. Such substances include acetamiprid and thiacloprid (Fig. 5). They are not adequate to be used as seed treatment because they quickly decompose. Acetamiprid toxicity for bees is low as it is easily metabolized by their organism (Iwasa et al. 2004).

On the world market, we can observe a continuous increasing use trend of neonicotinoids (Jeschke et al. 2011; Simon-Delso et al. 2015). Strategies of designing and synthesizing of cyclical neonicotinoids are even now a very attractive research field. For example, recent literature described divalent neonicotinoids, imidacloprid proinsecticides, neonicotinoids with N-substituted imine, bis-neonicotinoids, and crown-capped imidacloprid. Other studies showed that nitenpyram analogs with acyclic imine substitute show high biological activity (Yamamoto et al. 1998). These results show that cyclical skeleton is not an obligatory requirement for neonicotinoid to have insecticidal properties. It is useful to search continuously for new neonicotinoid compounds because it has been observed that their use too frequently in one given area, and for certain insect species, develops significant resistance. Modification of the structures of existing neonicotinoids may be an effective way of eliminating resistance in insects (Yang et al. 2014). Moreover, it was concluded that any insecticide leads to insect resistance when used continuously for a long period (Pisa et al. 2017). In the long term, such resistance cannot be overcome by developing new subgroups of insecticides, as demonstrated recently in the case of sulfoxaflor (Liao et al. 2017).

## Use of neonicotinoids

The last years made it clear that neonicotinoids are through frequently used insecticides for target pests like sucking insects, certain chewing insects, soil insects, and pests feeding on, e.g., grain crops, legumes, potatoes, and other vegetables, pomes, cotton, turf, etc. They are also used to kill fleas on house pets. Also, based on the action way, these insecticides became used worldwide due to some commercial marketing strategies (Simon-Delso et al. 2015). Neonicotinoids are useful for controlling sucking (e.g., aphids) and grubs that grow within the plant (e.g., wireworms), and can use the statement that they are selective to these groups of insects only. Table 2 presents information on some of these products, sold under different trade names, such as Gaucho, Cruiser, Confidor, and Actara, by various companies, e.g., Syngenta or Bayer. Neonicotinoids are systemic insecticides, which means that they do not remain on the plant’s surface, but enter its vascular tissue system and are transported through the whole organism. For this reason, part of these products is used into the treatment of seeds to provide the plant protection against pests from sowing time through germination until it is fully grown. Such total long-term protection remains active for a long time period and is distributed through stalks, leaves, and flowers of the plant, and then get into pollen, nectar, and the drops of water excreted by plant stomata (Bredeson and Lundgren 2018; Sur and Stork 2003).

**Table 2:** Examples of plant protection products available in Poland according to the regulation of the Polish Ministry of Agriculture and Rural Development (https://www.gov.pl/web/rolnictwo/rejestr-rodkow-ochrony-roslin)

Trade name*	Active substance (example concentration)	Description and applications
Cezar Hekplan Mospildate Shark Tenaz Vapcomore Mortal Profil Assail Intruder Tri-star Mospilan	Acetamiprid 20%	This is an insecticide that is a powder that should be dissolved in water. Type of action: contract, stomach Agricultural plants: potato, sugar beet, folder beet, winter oilseed rape, spring rape, tobacco. Fruit trees: apple tree, berry plants: raspberry, blackcurrant, strawberry. Vegetable plants (in ground and under cover): brassica, onion (from sowing and seedling), tomato, cucumber, paprika, eggplant. Ornamental plants (in the ground and under the covers).
Agroprim Gaucho Confidor Admire 2 Flowable Merit Provado Marathon	Imidacloprid 200 g/L	The formulation to combat flying insects (e.g., fly, mosquito, with the exception of wasps and hornets) and running insects (for example cockroach); indoors applications.
Clutch Poncho Dantotsu Fullswing Apacz	Clothianidin 500 g/kg (50%)	Insecticide in the form if granules to the water, suspension of operation of contact and distress; potatoes, apples, pears, gerbera
Actara Cruiser	Thiamethoxam 250 g/kg (25%)	It is an insecticide registered for the control of potato beetles and apple aphid and cactus cotton. Type of action: contact, stomach. Potatoes, apple trees.
Calypso Bariard Alanto	Thiacloprid 480 g/L (40, 40%)	It is an insecticide from the chloronicotinyl group, available as a water-soluble concentrate. The preparation is harmless to bees and many beneficial insects. Type of action: contact and stomach. Potatoes.
Proteus	Thiacloprid 100 g/L Deltamethrin 10 g/L	It is an insecticide with a systemic action against biting and stinging pests in general and vegetable crops. Type of action: contact, stomach. Potatoes, winter oilseed rape, sugar beet, maize.

Furthermore, imidacloprid may also be added to water used for hydrating plants. Formulations which offer controlled release of imidacloprid require between 2 and 10 days to release half of the active content in water (Adak et al. 2012). Neonicotinoids were also shown to remain for a long time in irrigation systems, drainage pipes, and soil. It was confirmed that these insecticides were still found at low levels in the soil even after 2 years from when the seeds were treated with neonicotinoids (Schaafsma et al. 2015). They were also present in the dandelion flower’s pollen (Krupke et al. 2012) and corn whose pollen was gathered by bees (Botías et al. 2017). Besides, the neonicotinoids such as imidacloprid, thiacloprid, and clothianidin were often used to treat soil as they have long periods of half-life in the earth (Bonmatin et al. 2015). For imidacloprid, this time is 26–229 days (Scorza et al. 2004) and even from 100 to 1,230 days (Baskaran et al. 1999). For clothianidin, even as many as 148–1155 days (about 5 months–about 38 months). The presence of two neonicotinoid insecticides—clothianidin and thiamethoxam—was detected in the body of dead bees and around beehives located near cultivated fields (Feltham et al. 2014). Some neonicotinoids are very persistent and they can remain for several years and accumulate in the surroundings due to repeated use, creating thus long-term risk (Bonmatin et al. 2015). Although the application doses for neonicotinoid insecticides are now much lower than for the previous generation of the commonly used organophosphates and carbamates, their harmful effects can still be noticeable even after a long time (Beketov and Liess 2008; Rondeau et al. 2014). As a consequence, this means greater exposure of pollinator insects because the neonicotinoids are present in all parts of a plant and during its whole growth (Goulson 2013). Besides plant protection products, sulfonamide neonicotinoids were found in different natural products and pharmaceuticals. Currently, the utilization of this neonicotinoids’ type is also increasing (Greenhill and Lue 1993; Yang et al. 2014).

## Modes of action and biological activity

**Fig. 6:**
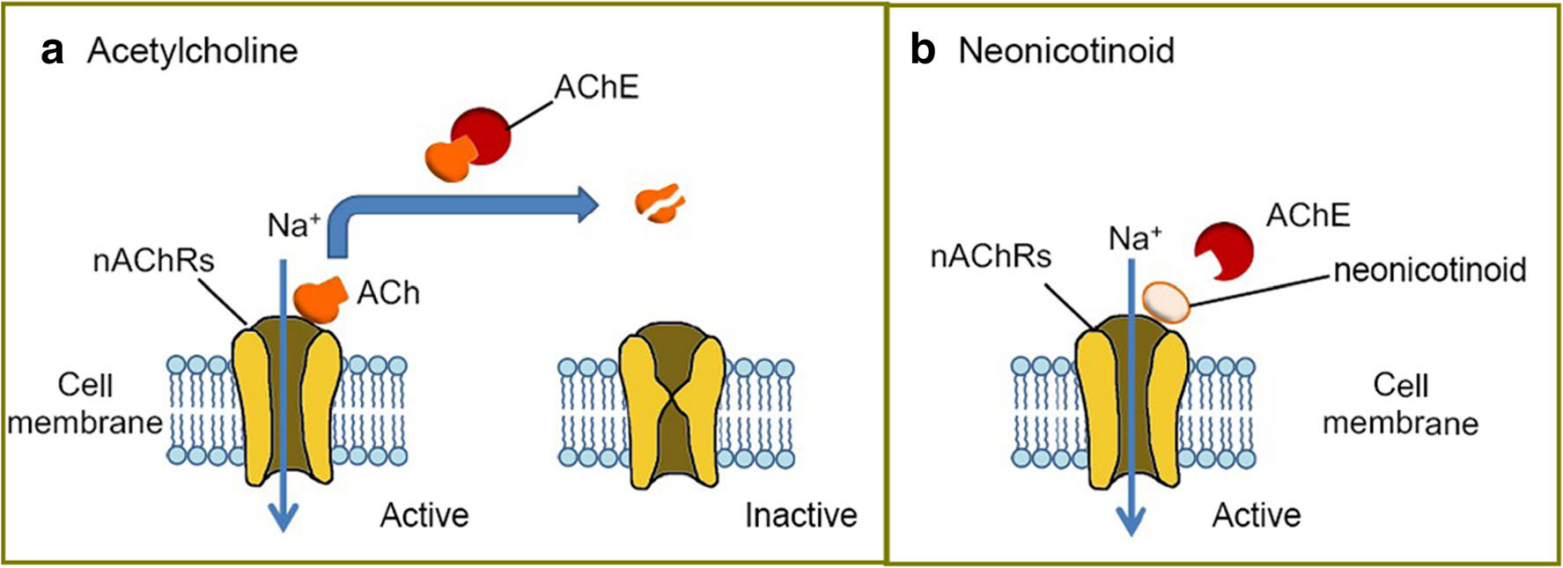
Schematic presentation of action of neonicotinoid acetylcholine receptors in the presence of acetylcholine and a neonicotinoid substance, according to ref. Chang et al. (2013)

Neonicotinoids are an important category of insecticides due to their effectiveness of action, the broad range of sucking insects and some grubs affected and reduced toxicity to mammals. These substances act selectively on the nicotine receptors of acetylcholine (nAChRs), which plays crucial role into the synaptic transmission in the central nervous system of insects (Casida and Durkin 2013; Kimura-Kuroda et al. 2012; Matsuda et al. 2001; Tomizawa et al. 2000; Wood and Goulson 2017; Yamamoto et al. 1995). Neonicotinoids are like nicotine agonists of nicotinic acetylcholine receptors (Casida and Durkin 2013; Kimura-Kuroda et al. 2012; Sheets et al. 2016). These receptors in mammals are located in the central and peripheral nervous systems, while in insects they are placed in the central nervous system. Most of neonicotinoids shows neurotoxic activity in the organisms of undesirable insects and binds much stronger to the neuroreceptors of insects than to those of mammals (Fig. 6) (Chang et al. 2013; Kimura-Kuroda et al. 2012).

Nevertheless, all animals with nervous system have nAChRs and neonics are agonists of the α4β2 (alpha-4 beta-2 nicotinic receptor) that make up these receptors. The difference between insects and vertebrates is that all nAChRs of insects contain these subunits, whereas in vertebrates only about 8–10% of nAChRs have them. The susceptibility of vertebrates to neonics, therefore, is much less than that of insects (Tomizawa and Casida 2005). To sum up this part of considerations is as follows: (i) the agonistic action on the α4β2 subunits, which elicits a neuronal impulse in the organism; (ii) the competition of neonic molecules with the natural neurotransmitter acetylcholine (Fig. 6); and (iii) the persistence of the stimulus, as the neonic is not deactivated by the enzyme acetylcholine-esterase, which leads to overstimulation and eventual death of the neurons (Chang et al. 2013; Rondeau et al. 2014).

Neonicotinoids act by binding no nicotine receptors of nAChRs and influence synaptic transmission. The receptors are usually activated by binding the neurotransmitter known as acetylcholine (ACh). They are subsequently deactivated when ACh is broken down by the acetylcholinesterase enzyme (AChE), producing octane and choline (Fig. 6a). Similar to ACh, neonicotinoids can bind to and activate nAChR, but—opposed to ACh—they cannot cause deactivation by AChE (Fig. 6b). This leads into the overstimulation of the nervous system and ultimately to cell death (Chang et al. 2013).

The places of binding neonicotinoids to nAChR are electronegative, which contributes to their toxicity in insects (Kimura-Kuroda et al. 2012; Yang et al. 2014). Due to this selectivity of the action of neonicotinoid insecticides, they were considered less toxic for mammals (Chen et al. 2014; Duzguner and Erdogan 2010). It revealed that neonicotinoids are usually present in food products. Although these levels were low, there appeared suggestions connecting health-adverse effects observed in honey with exposure to neonicotinoids. However, there is a growing amount of data proving that neonicotinoids (imidacloprid and clothianidin) have the ability to directly influence or change the activity of nAChRs in mammals (Chen et al. 2014; Kimura-Kuroda et al. 2012). Both in vivo and in vitro studies demonstrated that imidacloprid can change the membrane properties of neurons in mice; consequently, sensorimotor capability is significantly lowered and the level of glial lactic acid increases in the brain ventricle which is considered to be the center of emotions. Memory and autonomy of the nervous system was observed in newborn rats when the gravid female was exposed to trace amounts of such a substance. In most mammals, the undesirable neonicotinoids’ toxic effects are connected with their participation in binding to the α4β2 subunits of nAChR (Chen et al. 2014; Kimura-Kuroda et al. 2012). A study in vitro proved that imidacloprid and other neonicotinoids directly activate and change the α4β2 subtype of nAChR in humans. It is the best known subtype of nAChR in mammal brain, with the greatest density of receptors in diencephalon (*thalamus*). The α4β2 subtype of nAChR is implicated in a range of brain functions such as cognition, memory, and behavior. There are hard proofs regarding the role of α4β2 nAChR and the change in this receptor’s density in such CNS (central nervous system) disorders as Alzheimer’s or Parkinson’s disease, schizophrenia, and depression. During brain development (in utero), the α4β2 subunits of nAChR were involved in neuron proliferation, apoptosis, migration, cell differentiation, creation of synapses, and development of neuronal nervous systems (Chen et al. 2014; Sobkowiak and Lesicki 2011). It is likely that neonicotinoids can affect these processes during nAChR activation. Additionally, research in absorption using human intestinal cell line showed that neonicotinoids may be absorbed through membrane transport. Therefore, a question arises: can neonicotinoids potentially pose a danger to human health? Considering contemporary research and the scattered use of neonicotinoids in cultivation of plants and vegetables as well as their presence in foods, as well as still limited knowledge regarding toxicological repercussions of neonicotinoids on mammals, conducting epidemiological research is reasonable (Chen et al. 2014; Gibbons et al. 2015).

The mortality after the use of neonicotinoids for some insects and crustaceans has been taken into account. It is true that this class of compounds also interfere with the immune system and reproduction, with the possibility of significantly reducing the arthropod population in the environment, both in the terrestrial and in the aquatic (Pisa et al. 2017; Ruckert et al. 2018). The influence of neonicotinoids on aquatic organisms, in fact non-target organisms, cannot be overlooked (Basley and Goulson 2018; Hladik et al. 2018; Miles et al. 2017; Ruckert et al. 2018). The potential threat of neonicotinoids to wetland communities has been evaluated by experiments which simulated the exposures of invertebrates. The high tolerance to this group of compounds even the highest dissolvable concentration of insecticides was observed for freshwater snails and amphibian larvae with no mortality (Miles et al. 2017).

## Neonicotinoids’ impact on bees

Initially, neonicotinoids were considered to be substances that protect plants from pests but show low toxicity towards many useful insects (Blacquière et al. 2012; Tapparo et al. 2012; Lundin et al. 2015; Wood et al. 2018). However, this conviction has been recently put under question mark because of the great losses suffered by bee population all over the world. The mentioned phenomenon was called bee colony collapse disorder (Gliński and Kostro 2007; Neumann and Carreck 2010; Potts et al. 2010; vanEngelsdorp et al. 2009; van der Zee et al. 2012). The research studies proved that trace levels of neonicotinoids used in agriculture usually do not kill the bees directly (lethal effect) but have indirect (sublethal) influence as bees show all the symptoms of insecticide poisoning—uncoordinated movements, tremors, and convulsions. This affects the condition of bees, and their capacity to forage, learn, remember flower locations, and find the route back to the colony, hive, or swarm; it can also influence negatively the growth of honeybee and bumblebee colonies and the fertility of queens. This will not lead to sudden death of the insects but significantly worsens their health, disrupts nutrition processes, and impairs the sense of smell and navigational abilities; in consequence, the bees die sooner and more frequently. The observed sublethal effects include also physiological effects, i.e., developmental disorders in worker bees and queens (Cresswell et al. 2012; Decourtye et al. 2004; Laycock et al. 2012; Rumkee et al. 2017; Schneider et al. 2012; Tirado et al. 2013; Whitehorn et al. 2012;Yang et al. 2014).

To exemplify, the harmful influence of clothianidin involves immediate switching off of mitochondria in bee neurons while the negative impact of imidacloprid is visible only after longer exposure as it accumulates in the organism. Neonicotinoids disturb the functioning of insect nervous system including communication between brain areas, which can cause paralysis and death (Schneider et al. 2012). Furthermore, plant protection chemicals can lower the resistance of insects, including the bees and various pollinators to illnesses and parasites (Sánchez-Bayo et al. 2016). Simultaneous use of different classes of chemicals, including neonicotinoids and anti-parasite agents, also contributes to increased bee mortality (Fairbrother et al. 2014; Schneider et al. 2012; Tirado et al. 2013; Whitehorn et al. 2012).

**Fig. 7:**
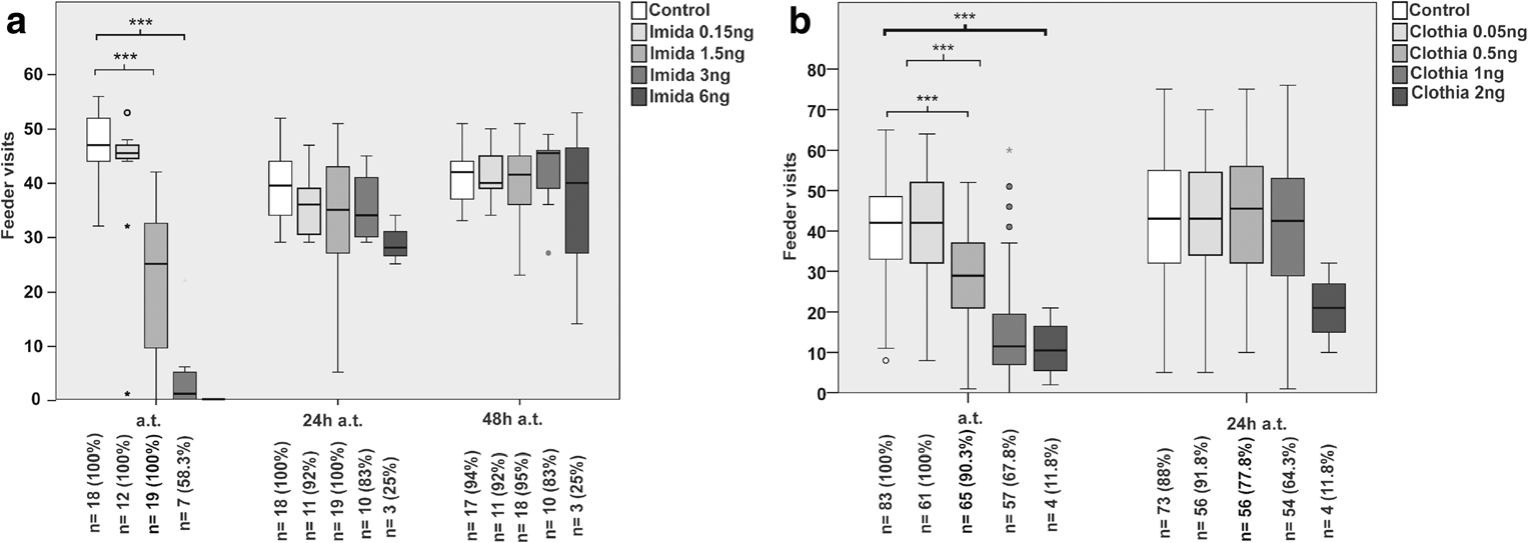
Frequency of bees leaving the hive after application of known doses of selected neonicotinoids (Imida, imidacloprid; Clothia, clothianidin). According to ref. Schneider et al. (2012)

**Fig. 8:**
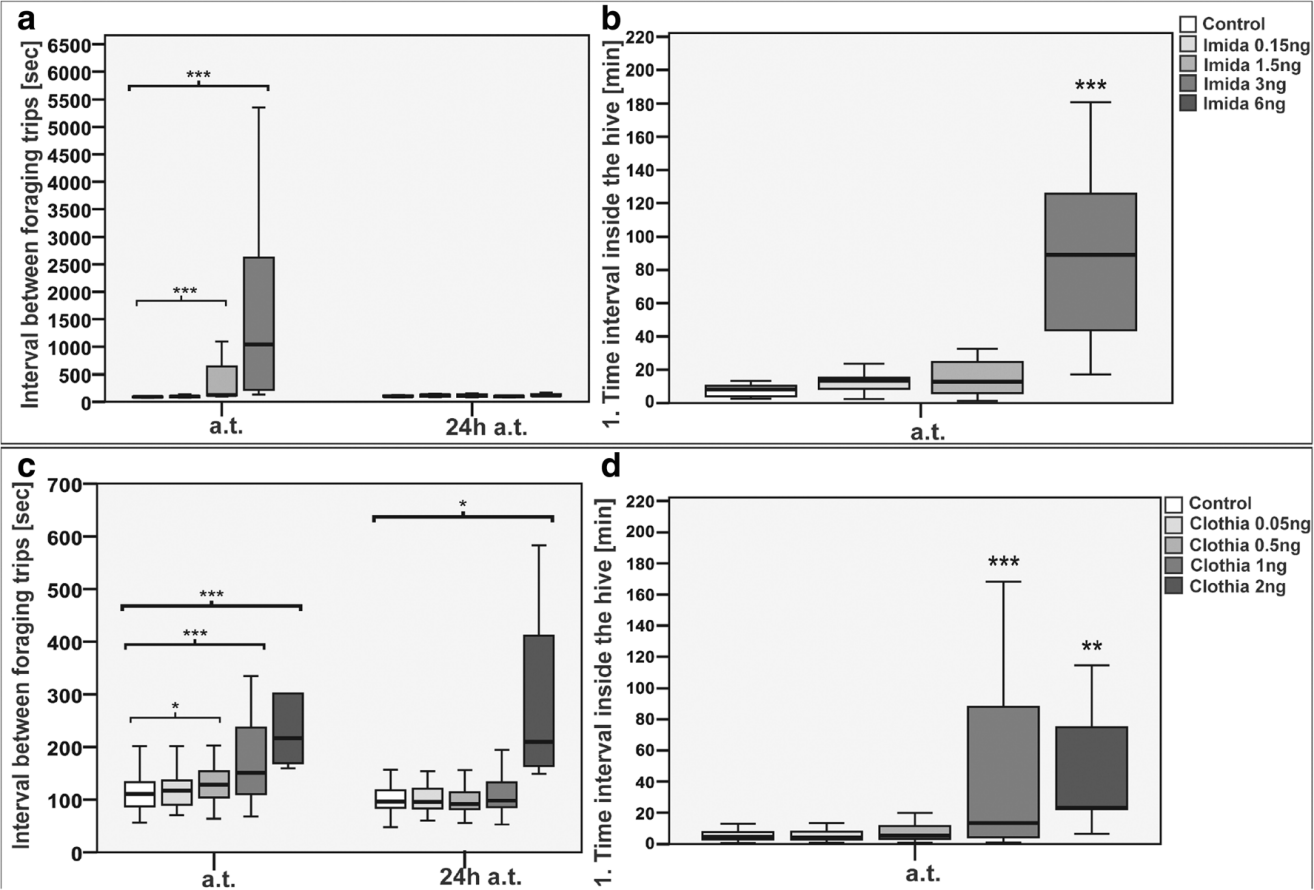
Changes in time between subsequent foraging flights of bees after application of known doses of selected neonicotinoids (Imida, imidacloprid; Clothia, clothianidin), according to ref. Schneider et al. (2012)

It is very worrying that bees prefer nectar contaminated by pesticides as consuming such food gives them more pleasure (Kessler et al. 2015). The bees also do not feel the taste of neonicotinoids, which increases the poisoning risk after eating contaminated nectar. The results of research on activity of bees exposed to different doses of imidacloprid and clothianidin can be observed in Figs. 7 and 8.

The fact is that neonicotinoids influence bees, similar with the fact that nicotine influences humans. The toxic effects of nicotine in humans are multiple, including sedative effects, whereas in insects are lethal. However, some insect species, for example a tobacco hornworm *Manduca sexta* L., is able to develop a much greater resistance to the toxic effects of nicotine (del Campo and Renwick 1999). It is supposed that they partially affect the insect brain which are responsible for preserving information regarding spatial orientation. Scientists are increasingly afraid that even trace amounts of pesticides can have negative influence on bees (Tennekes and Sánchez-Bayo 2011; Pisa et al. 2017). The conducted research unfortunately confirms these suppositions. Bees which were given clothianidin and imidacloprid showed less mobility in comparison with the control group (Schneider et al. 2012). In the case of the foraging flights, their number was lower and the time needed for gathering food was longer. Also returning time to the hive was longer and not all the bees returned. The staying time in the hive between flights was also extended. With the increase in the amount of neonicotinoid applied to the bees, the observed behavioral irregularities became more apparent. As clothianidin has lower contact toxicity in comparison with imidacloprid, its action is stronger, causing harmful effects even in lower doses. During the experimental proceedings, it was observed that these substances led into a significant reduction of foraging activity and to longer foraging flights at doses for clothianidin and imidacloprid of ≥ 0.5 ng/bee and ≥ 1.5 ng/bee respectively (Schneider et al. 2012).

## Ban on neonicotinoid use

Pollinator insects constitute a significant part of the environment. The last years revealed, however, that this balance has been significantly disrupted. Both agriculture and apiculture have more and more reasons to worry in connection with the threat to bees created by the effects of neonicotinoid pesticides (Sánchez-Bayo 2018). We cannot be sure that they are used in a safe way, in appropriate concentrations and at appropriate times. An answer to this issue is the Commission Implementing Regulation (EU) No. 485/2013 of 24 May 2013 amending Implementing Regulation (EU) No. 540/2011, concerning the approval conditions of active substances clothianidin, thiamethoxam, and imidacloprid, and prohibiting the use and sale of seeds treated with plant protection products containing those active substances (Commission Implementing Regulation 2013). The text was published in the Official Journal of the European Union, L 139/12, Vol. 56, May 25, 2013. Currently, the implementing regulations have been proposed by the European Commission (2018/783, 2018/784, 2018/785), in detail, which limited the use of these substances only for use in permanent greenhouses and for the treatment of seeds for the sowing only in such facilities. These regulations concern three neonicotinoids such as imidacloprid, clothianidin, and thiamethoxam. Nevertheless, the Polish Ministry of Agriculture has approved a derogation from the ban on the use of neonicotinoids in rape seed mortars. Also, a temporary permit for the use of mortars containing neonicotinoids to protect sugar beet has been issued. Note that many countries in Europe like Germany and France have introduced on their own prohibitions on the use of neonicotinoids.

## Selected analytical methods used for the determination of neonicotinoids

Neonicotinoids are used by farmers on a mass scale and frequently without any restraint, so it has become necessary to restrict the quantity of these substances in the products accepted as human food. Routine determination of neonicotinoids has been carried out only in the last several years, after it was enforced by decrees of the European Union. As new analytical procedures had to be performed in a relatively short time, the first methods used large amounts of toxic solvents (liquid–liquid extractions). The procedures were time-consuming and were based mainly on extracting analytes from samples by shaking, where a single use required up to 100 mL of solvent (e.g., dichloromethane).

Generally, for the determination of neonicotinoids separated from samples by liquid–liquid and/or liquid–solid extraction, the high-performance liquid chromatography (HPLC) is applied in most cases (Chen et al. 2014; Lehotay et al. 2010; Mandic et al. 2005). New methods use modern equipment and devices for isolating and determining the tested analytes, which increases the method sensitivity and shortens the analysis time. The main contributors are new extraction methods and liquid chromatography. HPLC detectors should be characterized by low sensitivity, high versatility as the ability of indication of a large number of substances, selectivity with respect to a certain number of substances, a wide linearity range, to work independent on the temperature and velocity of the mobile phase flow, and ease of use. Many detectors, i.e., UV/Vis, diode array detector (DAD), light scattering, corona discharge, fluorescence, conductivity, electrochemistry, radioactivity, and chemiluminescence, are available in various HPLC equipment, depending upon the application. Currently, various methodologies applied for the detection and quantification of the abovementioned compounds have been transformed by the use of automated liquid handling and HPLC tandem mass spectrometry detection techniques (LC-MS/MS).

The control of periodic changes in neonicotinoid concentration is included in the research program in many countries. An example can be investigations performed in the USA to control neonicotinoid pesticide residues in food (fruit, vegetable, meat, dairy, grain, honey, and baby food) and water samples. The investigation was continued for 16 years. Concentrations of neonicotinoid residues in food did not exceed those tolerance levels permitted by US EPA. However, higher concentration of imidacloprid has been found in water samples (untreated water) during 7 years from 2004 to 2011 (Craddock et al. 2019).

Moreover, this work presents selected procedures of determining neonicotinoids in crops and hive produce which will be suitable in the future for routine determination of contamination by residues of such substances. New methods use modern apparatuses and a variety of equipment both for determining and for isolating of the tested compounds, which has increased the sensitivity of the methods and shortened the analysis time. The main contributors here are new extraction techniques and detectors such as MS/MS. This part of the review presents selected separation methods which were verified on real-life samples and which are suitable for routine analyses of fruits and a variety of vegetables. The main object of testing for residual insecticides is food products that were most exposed to the contact with such substances, and processed food produced from such produce, e.g., juices. Imidacloprid was determined in fruit, fruit juices, and vegetables with LC-MS/MS or LC-DAD technique. Isocratic elution (Watanabe et al. 2007; Mandic et al. 2005) with water and acetonitrile (H_2_O/CH_3_CN) at the ratio 8:2 v/v or 75:25 v/v was used, and the packing of chromatographic columns were C_18_ sorbents (Hypersil ODS or Sun–Fire C_18_). If gradient elution was used, the mobile phase was 1% solution of HCOOH in water and 1% HCOOH in acetonitrile, and the solid phase was Phenomenex ODS (Lehotay et al. 2010).

The studies on the determination of neonicotinoid pesticides brought the publication of studies in which the researchers determined side by side several of the insecticides in use. Acetamiprid and thiamethoxam were determined in okra seed pods (*Abelmoschus esculentus* L.) (Singh and Kulshresta 2005); imidacloprid, acetamiprid, and thiacloprid were determined in different fruits and vegetables (Ortelli et al. 2004; Amelin et al. 2012) as well as in raisins (Hernandez et al. 2006). A different combination of pesticides was determined in vegetables: acetamiprid, nitenpyram, and imidacloprid (Obana et al. 2002). These procedures used only gradient elution with the solvents composed of H_2_O/CH_3_CN, sometimes additionally acidified with HCOOH. The detectors used were DAD (Amelin et al. 2012), MS (Hernandez et al. 2006), and MS/MS (Ortelli et al. 2004). Solid phases included Nucleosil C_18_HD, Lichrospher RP-18, Atlantis C_18_, and X Terra RP-18.

The studied analytes were isolated by extracting homogenized samples with ethyl acetate and rotating them (Ortelli et al. 2004) or with acetone, and subsequently, after salt precipitation and adding hexane, they were extracted with CH_2_Cl_2_ (Singh and Kulshresta 2005). In other studies, the targets were extracted with a mixture of methanol and water (80:20), and then was purified with the solid-phase extraction method (SPE) using OASIS HLB sorbent (Hernandez et al. 2006) or silica gel (Obana et al. 2002).

As the interest in the neonicotinoid analysis pollution was increasing, there appeared publications that described determining a larger number of these insecticides side by side, in different combinations. Acetamiprid, imidacloprid, thiacloprid, and thiamethoxam (Di Muccio et al. 2006; Wu et al. 2011) or acetamiprid, imidacloprid, thiacloprid, and nitenpyram (Ferrer and Thurman 2007) were detected in fruits and vegetables, and a mixture of clothianidin, dinotefuran, thiacloprid, and thiamethoxam—in various vegetables (Min et al. 2011). The pesticides were most frequently extracted with acetone, and the extracts were purified with the SPE technique using the sorbent Extrelux NT 20 ( (Di Muccio et al. 2006) or with QuEChERS sets well known as a quick, easy, cheap, effective, rugged, and safe sample preparation method (Wu et al. 2011; Ferrer and Thurman 2007; Min et al. 2011). Detection after gradient elution was carried out with MS (Di Muccio et al. 2006), DAD (Wu et al. 2011), TOF-MS (Ferrer and Thurman 2007), and MS/MS (Min et al. 2011) detectors. Solid phases were LiChrospher 100, Zorbax-Eclips XDB-C_8_, and Centurisil C_18_. The expanding use of mass spectrometers in detection is reflected in research covering an increasing number of neonicotinoids. This detection technique makes it easier to eliminate interference from other pesticides or sample matrix contents. Detection can also be performed now at lower concentration levels.

Five most often used neonicotinoids including acetamiprid, thiacloprid, thiamethoxam, and nitenpyram (Obana et al. 2003) or clothianidin, which is a replacement of nitenpyram (Benerjee et al. 2007), were identified in grapes, grapefruits, peaches, various vegetables, and rice. Extraction from plant samples was realized with either methanol or ethyl acetate; in the latter case, ultrasound was involved to support the process. Gradient elution was carried out with methanol and water (Obana et al. 2003) or methanol and water with the addition of 5 mM ammonium formate (Benerjee et al. 2007). In both cases, short (50 or 75 mm) chromatographic columns with C-18 packing were used.

In the mentioned study (Obana et al. 2003), the authors used an MS detector, achieving LOQ at the level of 0.01 mg/kg, while in another study (Benerjee et al. 2007) MS/MS detector was applied, which allowed the scientists to obtain a higher sensitivity for determined compounds (LOQ = 0.00025 mg/kg).

The subsequent publications bring the results of the research on the detection of a broadening range of insecticides. The studies determined in vegetable and fruit samples also imidaclotritz besides the five aforementioned insecticides (Zhang et al. 2012) or another combination of six substances: nitenpyram, clothianidin, thiacloprid, thiamethoxam, imidacloprid, and acetamiprid (Hiemstra and De Kok 2007).

In the study by Zhang et al. (2012), modified QuEChERs and LC-MS/MS techniques were developed. The method was validated for the analysis of 50 agricultural samples. Imidacloprid and imidaclotritz were detected at concentration levels between 5.3 and 7 μg/kg in real samples.

Moreover, the sixth mentioned pesticides, dinotefuran, was determined as a part of a 7-substance combination (Watanabe et al. 2007a; Liu et al. 2010). The UHPLC technique was used with a view to limiting the solvent volume and increasing the determination speed (Liu et al. 2010).

Other studies reported the determination of not only those pesticides, but also their metabolites. Clothianidin and its 4 metabolites in crown daisy, sedum, and amaranth grown in greenhouse conditions have been determined (Kim et al. 2012). The targets were identified and determined using LC-MS/MS. The LOQ were in the ranges of 0.04–0.16 mg/kg and they obtained recoveries between 71.7 and 120.3%. The methodology was successively used for the analysis of extracts that contain clothianidin and its metabolites in field-incurred samples (Kim et al. 2012).

The next analytical method (Rahman Md et al. 2013) was applied for dinotefuran and its metabolites in melon, using HPLC/UVD technique. For extraction and purification, the modified QuEChERs by acetate buffer was applied. The proposed method allowed for the obtaining of high water miscibility of some metabolites of dinotefuran, and lower sensitivity of UV detection in shorter wavelength was observed. The method was used for real samples, where dinotefuran and one of its metabolites were detected in the field-incurred melon samples. Residues were identified via LC-tandem mass spectrometry in positive-ion electrospray ionization (ESI (+)) mode.

Recently, due to the reports of possible threat that neonicotinoid insecticides pose to bees, the interest of researchers has been mainly focused on determining the residues of those compounds in bee products and the possible presence of neonicotinoids in bees themselves (Cicero et al. 2017; Feltham et al. 2014). Neonicotinoid poisoning of honeybees is a primary signal of negative effects of neonicotinoid application. The influence of nicotinoid insecticides and especially in the decline of bees is considered by several laboratories. Various researcher teams developed analytical methods for the separation, identification, and quantification of pesticides in honeybees and even other bee products like pollen, wax, and honey (Cicero et al. 2017; Feltham et al. 2014; Kasiotis et al. 2014).

Among the matrices of contaminating honeybees, a beebread can be considered. Some study was undertaken to develop and validate an original analytical approach that consisted on an extraction method based on modified QuEChERs, and by a selective analysis by UHPLC-MS/MS. The method was used for the analysis of 32 beebread samples and the presence of 7 of the target substances, and detected approximately concentration 170 ng/g for acetamiprid and thiacloprid. Although the contamination had low levels, this data indicates bees’ exposure to neonicotinoids via consumption of beebreads (Giroud et al. 2013). Martel and Liar (2011) describe the a multi-residue analytical method used for the identification of five neonicotinoids (imidacloprid, clothianidin, acetamiprid, thiacloprid, and thiamethoxam) in honeybees. The developed method was validated in detail. The extract in acetonitrile and n-hexane was analyzed by LC-ESI-MS/MS. The recovery data were obtained by spiking samples at two different concentrations of various neonicotinoids. For the experiments, honeybees without pesticides have been applied. The recoveries were between 93.3 and 104% (RSD< 20%), and LOQ = 0.5 ng/g for all pesticides, except acetamiprid, which was 1.0 ng/g. In another publication, for the presence of neonicotinoid residues in analyzed extracts, all samples from various areas of Greece were taken. Samples such as honeybees, bee pollen, and honey were taken into account. The total of 115 analytes among which neonicotinoids, organophosphates, thiazols, carbamates, dicarboximides, and dinitroanilines in honeybee bodies and honey and bee pollen were developed and validated by use of LC-ESI-MS/MS method. After the sample analysis, 14 active compounds were observed in all matrices, but for honey, only in one single sample the carbendazim was detected at 1.6 ng/g (Kasiotis et al. 2014).

The identification of neonicotinoids and their metabolites in honeybees and honey was achieved in the study (Gbylik-Sikorska et al. 2015). For the simultaneous analysis of pesticides such as imidacloprid, clothianidin, acetamiprid, thiamethoxam, thiacloprid, nitenpyram, and dinotefuran and their metabolites (in particular imidacloprid quinidine, imidacloprid olefin, imidacloprid urea, desnitro-imidacloprid hydrochloride, thiacloprid amid, and acetamiprid-*N*-desmethyl) in honeybee and honey, a new analytical method was developed. Preparation of honeybee samples involved extraction with acetonitrile and ethyl acetate and a cleaning up step using the Sep–Pak Alumina N–Plus Long cartridges. Extracts of honey were purified with Strata X–CW cartridges. The LOQs were between 0.1 and 0.5 μg/kg, and analyte recoveries ranged from 85.3 to 112.0%.

The next methods using LC-MS/MS for the determination of neonicotinoids were published in Kiljanek et al. (2016) and Cicero et al. (2017). Authors developed a method suitable for 200 pesticides and pesticide metabolites in honeybee samples. Proposed method was in detail validated. Metabolites of imidacloprid and thiacloprid have been detected. The sample preparation required the use of buffered QuEChERs method. Samples were extracted with acetonitrile containing 1% acetic acid and then cleaned up by dispersive solid-phase extraction using Z-Sep+ sorbent and PSA. The proposed method was developed to investigate more than 70 honeybee poisonings (Kiljanek et al. 2016). Cicero et al. (2017) described the monitoring of neonicotinoid pesticides in beekeeping. In that study, LC-MS/MS was applied and concentrations of neonicotinoids were determined in samples of honeybees, honeycomb, and honey, collected in 2015 during the blooming period from various areas in Sicily (Italy); the aim was to carry out an evaluation of bee product safety and to have an overview of neonicotinoid contamination in beekeeping. Obtained results made it possible to confirm only the presence of clothianidin in bee samples but these concentrations do not represent a risk for bees’ vitality.

Li et al. (2015) developed and compared two methods used for the analysis of selected pesticides in honeybees, pollen, and wax by GC/MS. Sample preparation methods consisting of solvent extraction as cleanup procedure performed by the gel permeation chromatography (GPC) and the dispersive SPE with zirconium-based sorbent (Z-Sep) were applied. Aims of investigation were the evaluation and comparison of matrix effects, method detection limits (MDLs), recoveries, and reproducibility of the analyzed pesticides. MDLs of the insecticides for the GPC method ranged from 0.40 to 8.30 ng/g dry weight, while MDLs for the Z-Sep method were from 0.33 to 5.37 ng/g dry weight. The recoveries ranged from 64.4 to 149.5% and 71.9 to 126.2% for the GPC and Z-Sep methods, respectively. It showed that the Z-Sep method is more appropriate for the determination of the target insecticides.

Recently, due to the reports of possible threat for bees posed by neonicotinoid insects, the research focuses mainly on determining the residue of such substances in bee products and their possible presence in bees themselves. In this part of the review of neonicotinoid determination methods, Table 3 presents selected separation methods, tested on real-life samples, which are suitable for routine analyses of a variety of fruit and vegetables; such bee products as pollen, wax, and honey; and bees themselves.

**Table 3:** Methods of neonicotinoid determination

Compound	Matrix	Sample preparation	Methods	LOQ recovery	Ref.
Imidacloprid	Fruit, fruit juice, vegetables	QuEChERS	LC-MS/MS LC-DAD	LOQ = 50 ng/g Recovery = 99–103%	Watanabe et al. 2007 Mandic et al. 2005 Lehotay et al. 2010
Acetamiprid, thiamethoxam	Okra fruits (*Abelmoschus esculentus* L )	Acetone, extraction, salting, hexane addition, next dichloromethane, and extraction	LC-DAD	n/a	Singh and Kulshresta 2005
Imidacloprid, acetamiprid, thiacloprid	Fruits, vegetables	Ethyl acetate, extraction	LC-MS/MS LC-DAD	LOQ = 0.01 mg/kg Recovery = 63–133%	Ortelli et al. 2004 Amelin et al. 2012
Imidacloprid, acetamiprid, thiacloprid	Raisin	Methanol:water (80:20) extraction, cleanup procedure by SPE, sorbents OASIS HLB	LC-MS/MS	LOQ = 0.01 mg/kg Recovery = 70–110%	Hernandez et al. 2006
Acetamiprid, nitenpyram, imidacloprid	Vegetables	Methanol:water (80:20) extraction, cleanup procedure by SPE, silica gel as sorbent	LC-DAD	LOQ = 0.2–2.0 mg/kg Recovery = 64–90%	Obana et al. 2002
Acetamiprid, imidacloprid, thiacloprid, thiamethoxam	Fruits, vegetables	Acetone, extraction cleanup procedure by SPE, sorbents EXTRELUX NT 20 or QuEChERS	LC-MS LC-DAD	LOD = 0.5–1 ng/g LOQ = 0.1–0.5 mg/kg Recovery = 74.5–105%	Di Muccio et al. 2006 Wu et al. 2011
Acetamiprid, imidacloprid, thiacloprid, nitenpyram	Fruits, vegetables	QuEChERS	LC-TOF-MS	LOQ = 10 μg/kg	Ferrer and Thurman 2007
Clothianidin, dinotefuran, thiacloprid, thiamethoxam	Vegetables	QuEChERS	LC-MS/MS	n/a	Min et al. 2011
Acetamiprid, thiacloprid, thiamethoxam, nitenpyram	Grapes, grapefruits, peaches, vegetables, rice	Methanol or ethyl acetate, extraction	LC-MS	LOQ = 0.01 mg/kg	Obana et al. 2003
Acetamiprid, thiacloprid, thiamethoxam, clothianidin	Grapes, grapefruits, peaches, vegetables, rice	Methanol or ethyl acetate, extraction	LC-MS/MS	LOQ = 0.00025 mg/kg	Benerjee et al. 2007
Acetamiprid, thiacloprid, thiamethoxam, clothianidin, imidacloprid	Fruits, vegetables	QuEChERS	LC-MS/MS	LOQ = 0.66–2.84 μg/kg Recovery = 73.7–103.8%	Zhang et al. 2012
Nitenpyram, clothianidin, thiacloprid, thiamethoxam, imidacloprid, acetamiprid	Fruits, vegetables	Acetone (dichloromethane/light petroleum extraction)	LC-MS/MS	LOQ = 0.01 mg/kg Recovery = 70–110%	Hiemstra and De Kok 2007
Nitenpyram, clothianidin, thiacloprid, thiamethoxam, imidacloprid, acetamiprid, dinotefuran	Agriculture samples Food—apple, cabbage, potato, tea, milk, pork, eggs	ChemElut SPE cartridge, acetone SPE cartridge packed with graphitized carbon	LC-DAD UHPLC-MS/MS	LOD = 0.01–0.03 mg/kg LOQ = 0.1–6 μg/kg	Watanabe et al. 2007 Liu et al. 2010
Clothianidin and 4 metabolites	Crown daisy, sedum, amaranth	QuEChERS	LC-MS/MS	LOQ = 0.04–0.16 mg/kg Recovery = 71.7–120.3%	Kim et al. 2012
Dinotefuran and metabolites	Melon	QuEChERS	UHPLC-UVD	LOQ = 0.06–0.16 mg/kg Recovery = 70.6–93.5%	Rahman Md et al. 2013
Neonicotinoids	Honeybees, pollen, wax, honey	QuEChERS	UHPLC-MS/MS	LOD = below ng/g Recovery = 53–119%	Giroud et al. 2013
Imidacloprid, clothianidin, acetamiprid, thiacloprid, thiamethoxam	Honeybees	Acetonitrile and n-hexane, extraction	LC-ESI-MS/MS	LOQ = 0.5 ng/g, an exception acetamiprid LOQ = 1.0 ng/g Recovery = 93.3–104%	Martel and Liar 2011
Neonicotinoids, organophosphates, thiazols, carbamates, dicarboximides, and dinitroanilines	Honeybees, bee pollen, honey	SPE	LC-ESI-MS/MS	LOQ = 0.03–23.3 ng/g Recovery = 59–117%	Kasiotis et al. 2014
Imidacloprid, clothianidin, acetamiprid, thiamethoxam, thiacloprid, nitenpyram, dinotefuran, some of their metabolites (imidacloprid quinidine, imidacloprid olefin, imidacloprid urea, desnitro-imidacloprid hydrochloride, thiacloprid amid, acetamiprid-*N*-desmethyl)	Honeybee, honey	Bees Mixture of acetonitrile and ethyl acetate, extraction, cleanup Sep–Pak Alumina N–Plus Long cartridges Cleanup of honey extracts by use of Strata X–CW cartridges	LC-MS/MS	LOQ = 0.1–0.5 μg/kg Recovery = 85.3–112.0%	Gbylik-Sikorska et al. 2015
Neonicotinoids and metabolites	Honeybee	QuEChERS, cleanup dSPE, Z-Sep+, and PSA	LC-MS/MS	LOQ = 1–100 ng/g Recovery = 70–120%	Kiljanek et al. 2016
Neonicotinoids and metabolites	Honeybees, honeycomb and honey sample	n/a	LC-MS/MS	n/a	Cicero et al. 2017
Pesticides	Honey bees, pollen, wax	Solvent extraction followed by gel permeation chromatography cleanup	gas chromatography-quadrupole mass spectrometry	MDLs = 0.40–8.3 ng/g Recovery = 64.4–149.5%	Li et al. 2015

## Conclusion

Bees and other pollinator insects are of immense importance for humans and for crop yield. Their presence in ecosystems brings huge benefits, estimated to amount to at least 153 billion euro on the global scale (22 billion euro in Europe itself). However, as bees pollinate the crops (Gallai et al. 2009; Garibaldi et al. 2014), they can be exposed to pesticides (Sánchez-Bayo and Goka 2014).

The demand for neonicotinoids has grown significantly in the last years. Fortunately, not all farmers use them, yet there are those who overuse these substances, applying them even when there are no pests. A drastic decrease is observed in the population of honeybees, which have permanent contact with neonicotinoids present at low levels in pollen and nectar coming from the neighborhood of cultivated fields.

Polish regulations permit the sale of a few plant protection products containing imidacloprid, clothianidin, acetamiprid, and thiacloprid. The issued permits a certain date of an expiration. Over the last 10 years in Poland, the sale of insecticide plant protection products has more than doubled. Sale and the use of insecticides have still grown in both cases, when mortars containing neonicotinoid were allowed and later after regulation. Therefore, the use of mortars containing neonicotinoid did not provide less use of insecticidal plant protection products or larger crops.

Based on the abovementioned investigations (Duzguner and Erdogan 2010; Schneider et al. 2012), it is worth it to ask a question: can neonicotinoids have a negative impact on the health of humans and other mammals, especially on developing brains?
